# Cinacalcet reverses short QT interval in Familial Hypocalciuric Hypercalcemia type 1

**DOI:** 10.1210/clinem/dgad494

**Published:** 2023-08-21

**Authors:** Thomas Cuny, Pauline Romanet, Michelle Goldsworthy, Carole Guérin, Marie Wilkin, Philippe Roche, Frédéric Sebag, Lynn E van Summeren, Mark Stevenson, Sarah A Howles, Jean-Claude Deharo, Rajesh V Thakker, David Taïeb

**Affiliations:** 1 Aix Marseille Univ, APHM, Marseille Medical Genetics, Inserm U1251, Hôpital de la Conception, Service d'Endocrinologie, Marseille, France; 2 Aix Marseille Univ, APHM, Marseille Medical Genetics, Inserm U1251, Hôpital de la Conception, Laboratoire de Biochimie et Biologie moléculaire, Marseille, France; 3 Nuffield Department of Surgical Sciences, University of Oxford, United Kingdom; 4 Aix Marseille Univ, APHM, Hôpital de la Conception, Service de Chirurgie endocrinienne, Marseille, France; 5 Aix Marseille Univ, APHM, Hôpital de la Timone, Service de Cardiologie, Marseille France; 6 Integrative Structural & Chemical Biology (iSCB) & HiTS Platform, Cancer Research Centre of Marseille, CNRS UMR7258, Marseille, France; 7 Academic Endocrine Unit, Radcliffe Department of Medicine, University of Oxford, Oxford, UK; 8 National Institute for Health Research Oxford Biomedical Research Centre, Oxford, UK; 9 Department of Nuclear Medicine, La Timone University Hospital, CERIMED, Aix-Marseille University, France

**Keywords:** FHH-1, cinacalcet, QT interval, hyperparathyroidism, CASR

## Abstract

**Context:**

Familial hypocalciuric hypercalcemia type 1 (FHH-1) defines an autosomal dominant disease, related to mutations in the *CASR* gene, with mild hypercalcemia in most cases. Cases of FHH-1 with a short QT interval have not been reported to date.

**Objective:**

Three family members presented with FHH-1 and short QT interval (< 360 ms), a condition that could lead to cardiac arrhythmias, and the effects of cinacalcet, an allosteric modulator of the CaSR, in rectifying the abnormal sensitivity of the mutant CaSR and in correcting the short QT interval were determined.

**Methods:**

*CASR* mutational analysis was performed by next-generation sequencing and functional consequences of the identified CaSR variant (p.Ile555Thr) and effects of cinacalcet were assessed in HEK293 cells expressing wild-type and variant CaSRs. A cinacalcet test consisting of administration of 30 mg cinacalcet (8am) followed by hourly measurement of serum calcium, phosphate, and PTH during 8 hours, and an ECG was performed.

**Results:**

The CaSR variant (p.Ile555Thr) was confirmed in all three FHH-1 patients and was shown to be associated with a loss of function that was ameliorated by cinacalcet. Cinacalcet decreased PTH by >50% within two hours, and decreases in serum calcium and increases in serum phosphate occurred within 8 hours, with rectification of the QT interval, which remained normal after 3 months of cinacalcet treatment.

**Conclusion:**

Our results indicate that FHH-1 patients should be assessed for a short QT interval, and a cinacalcet test used to select patients who are likely to benefit from this treatment.

## Introduction

Familial hypocalciuric hypercalcemia (FHH) is an autosomal dominant genetic disease mainly caused by germline calcium-sensing receptor *(CASR)* inactivating mutations (also called FHH-1)(1). More rarely FHH can be related to *AP2S1* or *GNA11* inactivating mutation, both genes encoding for proteins involved downstream of *CASR* activation(2,3). FHH is usually characterized by mild elevation of calcemia with inappropriate or normal plasma parathyroid hormone (PTH) and, eventually, very low 24-hour urinary calcium excretion, best expressed by a urine calcium-to-creatinine clearance ratio (CCCR) <0.01(3). Parathyroid thyroid imaging, if done, can be negative, a finding that together with hypocalciuria should raise the suspicion of FHH and argued against parathyroidectomy(4).

Hypercalcemia can be complicated by cardiac arrhythmias, as a consequence of a short QT interval(5). To date there is no data reporting the occurrence of possible cardiac arrhythmias in FHH-1 patients, possibly linked to the mild biochemical phenotype usually observed in this context.

Cinacalcet, a positive allosteric modulator of CaSR, can decrease PTH secretion(6) and was successfully used for FHH with a more severe hypercalcemia(7,8), however its benefits in mild presentation of FHH is counterbalanced by possible side effects (hypocalcemia, nausea, vomiting) and cost burden (9,10). In certain circumstances, the use of cinacalcet can be discussed on an individual basis at a multidisciplinary team meeting to ensure proper indication and follow-up. This can be the case in patients with short QT interval, a finding that could predispose to sudden cardiac death(11). Here, we present three family members with FHH-1 and a severe short QT interval who were potential candidates for cinacalcet therapy after demonstrating their sensitivity to the compound via a cinacalcet test.

## Methods

### Study medication

Cinacalcet (Amgen®, Thousand Oaks, Cal, USA) is a small molecule positive allosteric modulator of CaSR, which potentiates ionized free extracellular calcium (Ca^2+^i) activity at the CaSR. As such, cinacalcet left-shifts the Ca^2+^i/PTH concentration-response relationship in the body, meaning that lower Ca^2+^i concentrations are required to suppress PTH release(6). Cinacalcet is FDA approved for the treatment of PHPT in patients who cannot undergo parathyroidectomy, in adults with parathyroid carcinoma, and in patients on renal replacement therapy with secondary hyperparathyroidism. Main adverse effects described are hypocalcemia and gastrointestinal side effects (nausea and vomiting), but recent data are reassuring on the occurrence of these side effects(12). In adults, it is common to start by dose of 30 mg per day and then to further adapt on the tolerance and the calcium level.

### Patients’ phenotypes and genotypes

We report here three FHH-1 family members, one mother and her two children (one son, one daughter), caused by germline mutation of *CASR*, with the latter two being treated by cinacalcet for short QT interval (Table 1).

The mother was the index case and was initially misdiagnosed as primary hyperparathyroidism (PHPT) and operated. At diagnosis, her ECG showed a moderate short QT interval of 360 ms. She developed persistent hypercalcemia following resection of two hyperplastic parathyroid glands. After surgery, a heterozygous pathogenic variant in the *CASR* gene (NM_000388: exon 6, c.1664T>C, p.Ile555Thr, I555T) was identified by next generation sequencing and then confirmed by targeted Sanger sequencing (13). After informed consents were obtained, a genetic screening of her two children revealed that they carried the same *CASR* pathogenic variant. The father of both children did not have the *CASR* mutation.

**Figure 1:**
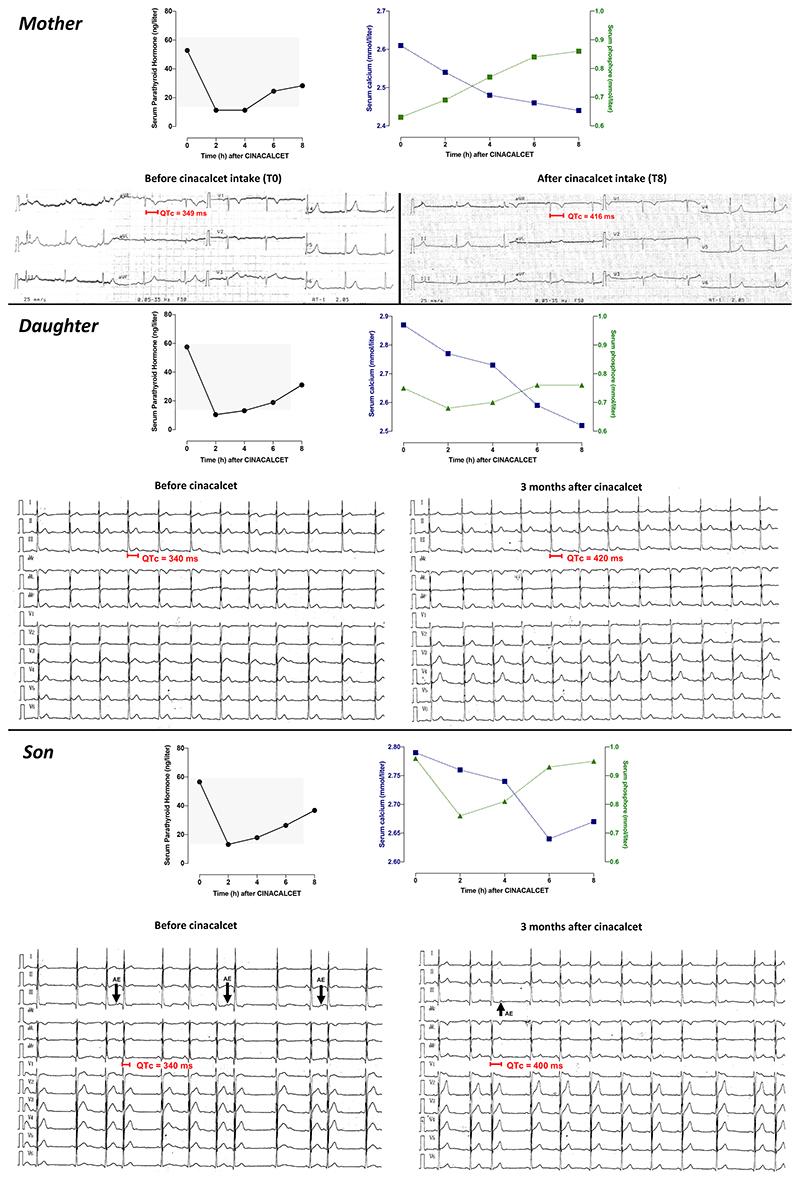
Cinacalcet test and Electrocardiograms of FHH-1 patients before and during Treatment. Functional test assessing the sensitivity to cinacalcet (30mg) of parathyroid hormone secretion, serum calcium and phosphate over an 8-hour period. In addition, ECG was performed in the mother at baseline (T0) and at the end of the test (T8) and showed a normalization of the QT interval following the intake of cinacalcet. In children, ECG before treatment and after 3 months of cinacalcet are presented. They are characterized by short QT interval, in the daughter and atrial extrasystoles (AE) in the son. Cinacalcet therapy normalized QTc in both patients.

Her daughter was aged 20yrs at diagnosis with a past medical history of syncope at 16-yrs while practicing sport but did not have any further investigations. She only complained of palpitations (even during rest) during 2 to 3 minutes, occuring 3 times monthly. She had a marked hypercalcemia (2.95 and 2.87 mmol/l) with hypophosphatemia (0.75 mmol/l, normal range: 0.81 - 1.45), normal high PTH 1-84 (57.5 pg/ml, NR 15.1-65.1), low vitamin D level (35 nmol/l, NR: 75 - 250), and CCCR<0.01. Albumin and magnesemia were in the normal range. Parathyroid ultrasound and scintigraphy were negative. ECG revealed a short QT interval of 340 ms (corrected with Bazzet’s formula) (Fig. 1).

Her son aged 19-yrs had no past medical history and presented with an asymptomatic hypercalcemia (2.88 mmol/l and 2.85 mmol/l). Serum phosphate was normal (0.96 mmol/l), PTH was in the upper range limit (56.6 pg/ml), vitamin D was low (31 nmol/l) and CCCR was decreased (0.006). ECG showed atrial extrasystoles and a short QT interval (340 ms).

Testing all the family members for mutations of *KCNH2*, *KCNQ1*, *KCNJ2*, *CACNA1C*, *CACNB2* and *CACNA2D1* to identify a genetic cause of the short QT interval syndrome, did not identify any pathogenic variants. Other secondary causes of a shortened QT interval were also absent (hyperkaliemia, acidosis, hyperthermia, intake of drugs, e.g. digoxin)(5). Transthoracic echocardiography was normal in the 3 cases.

A multidisciplinary meeting confirmed that the ECG modifications could be the source of severe heart arrhythmias with a risk of sudden death. Because oral hyperhydration was ineffective in controlling hypercalcemia in the patients, a decision to treat these patients with calcimimetic was taken after performing a functional test to ensure a response to the treatment.

### DNA sequencing

Genomic DNA was extracted with QiaSymphony DS DNA Midi Kit (Qiagen, Courtaboeuf, France) from blood lymphocytes (standard EDTA samples). Exons and 20 bp flanking introns of *CaSR* (HHF1, NM_000388) were sequenced by next-generation sequencing (NGS) using a QiaSeq custom library (Qiargen, Courtaboeuf, France) according to the manufacturer’s instructions. Libraries were sequenced on MiSeqDX (Illumina). The alignment and variant calling were performed using the Biomedical Genomics Workbench 5.0.1 software (Qiagen) and based on human genome GRCh37/hg19. Each variant was classified according to the guidelines of the American College of Medical Genetics and Genomics.

### In silico homology modeling

Human CaSR is expressed on the cell surface as a constitutive homodimer(14). The dimerization plays a key role in the activation of the receptor upon calcium binding leading to large conformation changes and rearrangements(15). Three-dimensional homology models were built for I555T mutant of human CaSR (Uniprot P41180) with MODELLER software v9.24(16). The recent 3D structure of wild type human CaSR in the active form was used as a template (PDB code 7DTT)(15). Ten 3D models were generated and the one with the best objective function was selected for illustration. *In silico* modeling predicted that the I555T missense mutation disrupts Ca^2+^ signalling by altering the twisting movement of the loop of the Cysteine-rich Domain (CRD) upon activation and the Ca^2+^ binding.

### Generation of CASR Ile555Thr Expression Construct

A pcDNA5/FRT construct (ThermoFisher Scientific) containing a full length human *CASR* cDNA was mutagenized using the QuikChange Lightning Site-Directed Mutagenesis kit (Agilent Technologies) and *CASR* specific primers (Forward - 5’ CTC CCC CTCAGT GATCCC TTT CCT GGT CCC; Reverse - 5’ GGG ACC AGG AAA GGG ATC ACT GAG GGG GAG; Thermo Fisher Scientific). Mutagenesis was analyzed in eight selected clones by PCR followed by Sanger sequencing using *CASR* specific sequencing primers (Forward - 5’ GTG CAG ACA TCA AGA AAG TTG AG; Reverse - 5’ GGT GTA GAG CCA GAT CAC AC). To ensure no other mutations had been introduced, sequencing of the entire *CASR* coding region using *CASR* specific sequencing primers (FP1 – 5’ CAA CAC CGT TTC TAA GGC C; FP2 – 5’ GTG CAG ACA TCA AGA AAG TTG AG; FP3 – 5’ GGA AGC TGC CGG AGA AC; FP4 – 5’ GTC ATC TTT GGC AGC GGC; RP1 – 5’ CTT GCT GCT GAT GGA GG; RP2 – 5’ GGT GTA GAG CCA GAT CAC AC; RP3 – 5’ CAT CCC CTG TAC AGA GGG; RP4 – 5’ CGG CCT TGA TTT GAG ATC TTG) was performed.

### Cell culture and transfection

Functional studies were undertaken using HEK293 cells. Cells were plated in 96 well plates and transfected with 50ng per well wild-type or Ile555Thr pcDNA5/FRT-*CASR* using 0.1μl per well lipofectamine 2000 (Thermo Fisher Scientific) 48 hours prior to experiments and maintained in DMEM-Glutamax media (Thermo Fisher Scientific) with 10% FBS (Gibco) at 37°C, 5% CO_2_.

### Western blot analysis

Protein was extracted 48 hours post transfection in Pierce RIPA Buffer (ThermoFisher Scientific) and Western blot analyses were undertaken using anti-CaSR (5C10, ADD; ab19347; Abcam), and anti-α-Tubulin (ab15246, Abcam) antibodies. Western blots were performed in un-transfected HEK293 and HEK293 cells transfected with wild-type or Ile555Thr pcDNA5/FRT-*CASR* and visualized using an Immuno-Star Western C kit (Bio-Rad) on a Bio-Rad Chemidoc XRS+ system.

### Measurement of intracellular calcium responses

At 48 hours post transfection, cells were washed with 100μl Complete Imaging Buffer (150mM NaCl, 2.6mM KCl, 1.18mM MgCl_2_, 10mM HEPES, 0.1mM CaCl2, pH 7.4), loaded with Fluo-4 dye (Complete Imaging Buffer supplemented with; 1μM Fluo-4 AM, 0.01% pluronic F-127, 0.5% BSA), and incubated for 60 min at 37°C. Cells were washed again before the addition of a further 100μl of Complete Imaging Buffer with the addition of either 100nM Cinacalcet (AMG-073 HCL) in DMSO or vehicle only control and incubated for 30 minutes at room temperature in the dark. Using an automated system, calcium chloride was injected into wells to achieve extracellular calcium concentrations ranging from 0.05-1.5mM. Control wells were injected with 10μM ionomycin. Changes in intracellular calcium concentrations were recorded via detection of fluorescence for 30 seconds using a PHERAstar microplate reader (BMG Labtech) at 37°C with an excitation filter of 485 nm and an emission filter of 520 nm. The peak mean fluorescence ratio of the transient response following each individual stimulus was measured using MARS data analysis software (BMG Labtech). Relative fluorescence units were normalized to the fluorescence stimulated by ionomycin to account for differences in cell number and loading efficiency, data was expressed as a percentage of wild type CaSR maximal response. Assays were performed using 9 biological replicates (independently transfected wells, performed on at least 9 different days). Nonlinear regression of concentration-response curves and 2 way ANOVA tests with Sidak’s correction for multiple testing was performed in GraphPad Prism.

## Results

### CaSR Variant classification criteria

The c.1664T>C, p.Ile555Thr variant identified in the 3 family members, is absent from controls (gnomAD v2.1.1 and gnomad v3.1.2, last access 11/03/2023). It was reported 6 times in literature (4,17–21) in patients with, or suspected to present FHH, and was always considered as pathogenic or likely pathogenic. It was published once at the heterozygous state in an infant with neonatal severe hyperparathyroidism. There is one other variant reported in literature on the same amino acid position (c.1663A>G, p.Ile555Val) in a patient suspected to present FHH, but no information was available on patient’s phenotype or variant classification (22).

Segregation studies showed co-transmission of genotype and phenotype in literature (21) (7 family members) and in two unrelated families analyzed in our lab (this family, 3 family members, and an unreported second family with 8 family members).

This variant is located in the extracellular domain of the protein, in the cysteine-rich region, close to the membrane, that is highly conserved. The nucleotide (located in Chr3(GRCh37):122001015T>C) is moderately conserved (phyloP: 4.98 [-19.0, 10.9]), but comparison between the species shows a highly conserved amino acid (Ile present in 11/12 species) (13). There is a moderate physicochemical difference between Ile and Thr (Grantham dist: 89 [0-215]), but *in silico* analysis predicted a pathogenic effect (Revel: 0.689; PolyPhen2 HDivPred: probably damaging (score: 0.984), HVarPred: possibly damaging (score: 0.855); MutationTaster (v2021): Deleterious. Tree vote: 95|5, SIFT(v6.2.0): DELETERIOUS(score: 0.04, median: 4.32)), except for Align GVGD (v2007): Class C0 (GV: 135.42 - GD: 0.00) (13) The *CASR* is a gene with low rate of benign missense variants (Z score constraint metrics for missense variants 3.12 gnomADv2.1.1) and with numerous known pathogenic missense variants (literature).

### Structural modeling

Residue 555 of CaSR is located at the dimer interface and mutation of the hydrophobic isoleucine to polar threonine may disrupt the dimerization process. The loop of the CRD domain close to LB2 containing residue 555 undergoes a significant twisting movement upon activation of CaSR(15). This movement could be altered when I555 is mutated into a threonine. In addition, residue 555 is located near the second Ca^2+^ binding site (between LB2 and CRD domains) and I555T mutation could directly or indirectly disrupt Ca^2+^ binding to this site. Of note, mutation of a nearby residue, Gly555Glu (G557E) completely abolishes Ca^2+^ binding. Finally, I555T mutation could influence receptor dynamics and communication between domains LB1/LB2 and TMD domains (via the Cystein-rich Domain) (13). To illustrate the key position of the residue 555 in the activation of CaSR, a movie was generated and added as a supplementary motion (13).

### Functional analysis CaSR Ile555Thr variant

**Figure 2:**
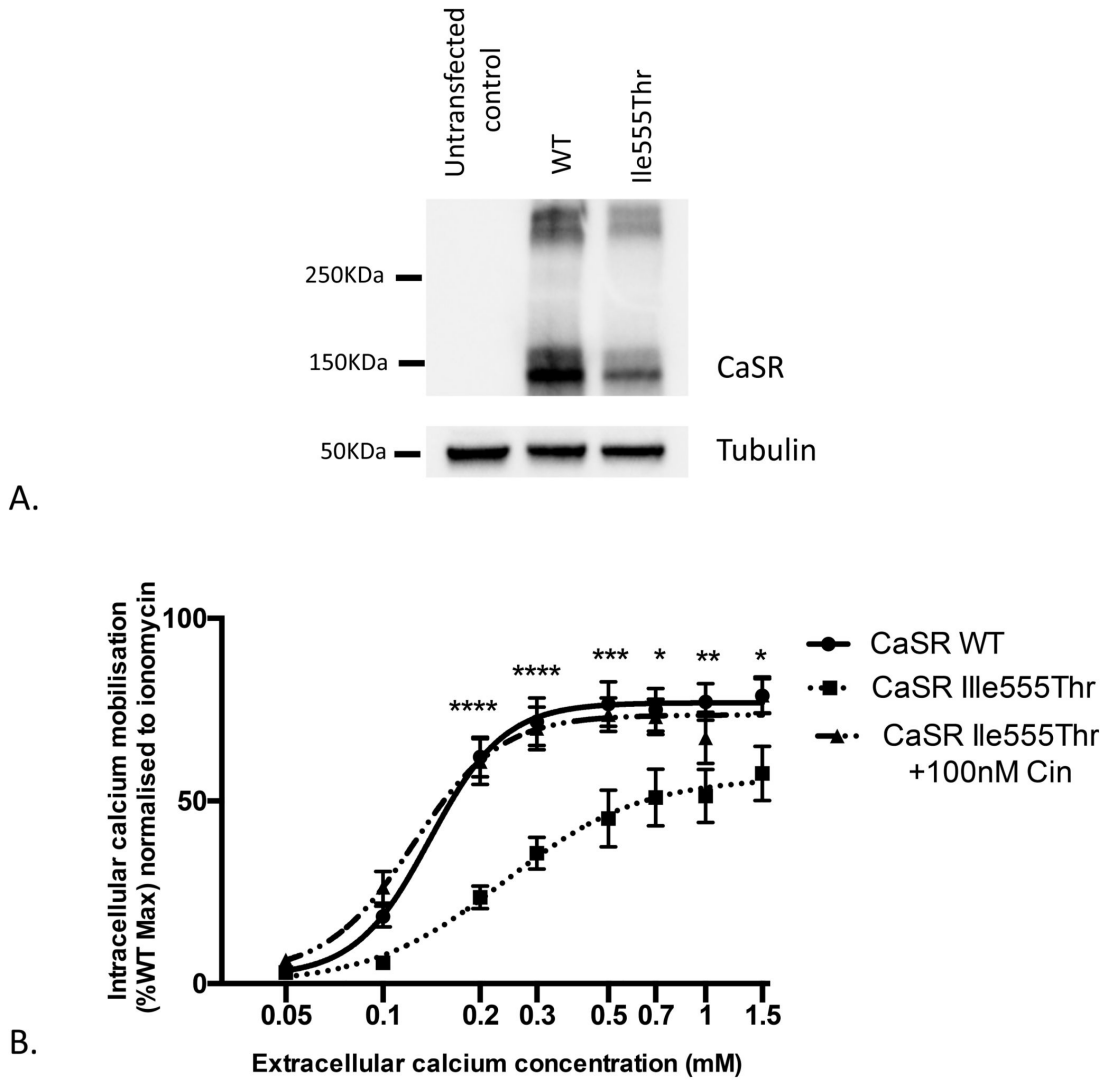
A) Western blot analysis demonstrating comparable expression of wild-type (WT) and Ile555Thr CaSR proteins. B) Intracellular calcium responses to changes in extracellular calcium concentrations in HEK-CaSR cells transiently transfected with either wild-type (WT, solid circles) or Ile555Thr (solid squares) *CASR*. Addition of 100nM cinacalcet to cells expressing Ile555Thr CaSR protein rectified the rightward shift in concentration-response curves (filled triangles). Ca^2+^_i_ responses are expressed as a percentage of the maximum normalized WT response and shown as the mean (±SE) of 9 assays from 9 independent transfections. Comparison of WT and Ile555Thr responses *p<0.05, **p<0.01, ***p<0.001, ****p<0.0001.

We hypothesized that the Ile555Thr variant would result in impaired CaSR function and hypothesized that this mutation would result in a decrease in the sensitivity of cells expressing Ile555Thr CaSR to extracellular calcium. To investigate this hypothesis, HEK293 cells were transfected with wild-type or Ile555Thr pcDNA5/FRT-*CASR* expression constructs (13). Western blot analysis demonstrated comparable expression of wild-type and Ile555Thr CaSR; immature glycosylated monomer (140KDa), mature fully glycosylated monomer (160KDa) and dimeric (250KDa) bands were present at similar levels (Figure 2 A). Expression of Ile555Thr CaSR resulted in a reduction in intracellular calcium responses to alterations in extracellular calcium concentrations, with a rightward shift in the concentration-response curve consistent with a loss-of-function mutation in the CaSR. Cinacalcet, at a concentration of 100mM was able to correct this abnormal intracellular calcium signaling (Figure 2 B).

### Acute cinacalcet Test

The test was performed in the daily hospital unit. Patients provided informed written consent. Cinacalcet (30 mg) was orally given to the patient at 9am and serum calcium, phosphate and PTH levels were assessed every 2 hours for 8 hours. In all 3 patients, administration of cinacalcet resulted in >50% decrease of PTH 1-84 within the first two hours (Figure 1). Decrease in serum calcium occurred within the 8 hours following cinacalcet intake in the 3 patients, whereas an increase in serum phosphate occurred in the mother and her son. Moreover, ECG performed in the mother at baseline (T0) and at the end of the test (T8) showed a normalization of the short QT interval from 349 ms to 416 ms (Fig. 1).

### Biochemical and Electrocardiographic outcomes under Cinacalcet therapy

Following the positive cinacalcet tests, the treatment was proposed in all 3 patients and initiated at 30mg twice a day with monthly biochemical measurements. The mother had nausea even with the lowest dose, and she had to discontinue the treatment due to side effects. Subsequently, her QT interval returned to 360 ms. In her daughter, cinacalcet was well tolerated and led to normalization of the plasma calcium to 2.25, 2.4 and 2.47 mmol/l at month 1, 2 and 3, respectively (Table 1). PTH decreased to 32, 57 and 43 pg/ml, at the same study points. QTc interval normalized to 420ms after 3 months of treatment with disappearance of early repolarizations (Figure 1). In the son, cinacalcet resulted in decrease of both plasma calcium to 2.65, 2.6 and 2.55 mmol/l, and PTH to 13, 13 and 27 pg/ml, at month 1, 2 and 3, respectively. On the ECG, QTc interval returned to normal value (400ms) with a decrease in atrial excitability as shown by reduction of atrial extrasystoles.

### Safety and adverse events

No side effect was observed during the acute cinacalcet test. On the long-term treatment, the mother had nausea with cinacalcet, that persisted despite reduction in dosage, the reason why the mother preferred to stop the treatment. On the contrary, no adverse effects such as nausea, vomiting, and hypocalcemia occurred during cinacalcet treatment in her two children.

## Discussion

Hypercalcemia represents one of the most frequent metabolic disorder encountered in clinical practice (prevalence of up to 1/1000)(23). The regulated flux of Ca^2+^ within and through cardiomyocytes governs both cardiac excitability and contractility by influencing the activity of voltage-dependent calcium channels (VDCC) which play a critical role during phase 2 of action potential in the cardiomyocyte (24). Hypercalcemia changes the shape of the ventricular action potential into that of an atrial action potential, shortening the duration of phase 2, whose electrocardiographic translation is shortening in the QT interval and shortening or absence of the ST segment (25). Previous studies showed that CaSR was expressed in cardiomyocytes, especially in those from the left-ventricular myocardium, where it contributed to exert membrane-stabilizing effects (26). The latter is further suggested by the anti-arrhythmic properties of putrescine, a natural ligand of CaSR (27). Whether or not mutated CaSRs favor arrhythmias, is currently unknown. In the cardiomyocytes, calcium likely functions as both first and second messenger and as such activated CaSR will influence both the contraction and the relaxation of the cardiomyocytes. These mechanisms depend on Gαq/11 pathways involving inositol trisphosphate (IP3)-dependent and independent pathways (26,28). Through the effect of IP3, Ca^2+^ is released from the sarcoplasmic reticulum which results in an improvement in the cell shortening by increasing the systolic calcium. Gαq/11 also leads to the activation of the protein kinase C (PKC) (by generation of diacyl glycerol), which will lead to pump calcium back into its intracellular storage. As such, the diastolic calcium concentration remains stable, resulting in an increase in the relaxation speed of the cells (29).

Therefore, it is likely that calcimimetics, like cinacalcet, may modulate the electromechanical coupling of cardiomyocytes by 1) decreasing ionized calcium concentration, that, in turn, will influence the activity of VDCC (the “indirect” effect) and 2) by binding the CaSR expressed at the surface of cardiomyocytes (the “direct” effect). Currently, the impact of mutated CaSRs in the heart, as in syndromic condition like FHH-1 is unknown. FHH-1 was classically considered as benign, but a recent study reported that loss of function *CASR* variants were associated with altered risks of common diseases including cardiovascular diseases(30). By analogy, the occurrence of short QT or ventricular premature beats in PHPT is still debatable (31). Previous studies suggested that PHPT increases the risk of arrhythmias (32,33) while another study did not support this finding(34). However, the latter included patients with moderate hypercalcemia (median serum calcium of 2.67 mmol/l (IQR 2.60-2.77) and minor ECG abnormalities (mean corrected QT interval of 414 ± 24 ms).

We describe here three family members with FHH-1 who exhibited chronic marked hypercalcemia and baseline QTc interval below 360ms, suggesting that these metabolic conditions could increase the risk of cardiac arrhythmias and sudden cardiac death (SCD). Of interest, previous study showed that in a patient with an autosomal dominant hypocalcaemia due to constitutive activation of CaSR, QT interval was found to be excessively long (> 440 ms) (35). Short QT interval is listed as a cause of SCD but has been extensively studied in the setting of congenital short QT syndrome (SQTS)(5) and to the best of our knowledge, there is no other clinical cases that report short QT interval in either FHH-2 or FHH-3 patients. The link between short QT interval and SCD in acquired causes of hypercalcemia like PHPT or FHH, is, therefore, currently unknown. Still, we keep in mind that the daughter in this family had a past medical history of syncope at 16-yrs.

Here, we show that cinacalcet normalized QT interval in young FHH-1 patients who are at risk of heart conduction disorders. Whether discontinuing cinacalcet leads to a relapse of short QT in these patients, remains to be established. Most FHH-1 patients present a milder clinical and biochemical phenotype and the benefit of cinacalcet can be discussed considering the potential cost burden and side effects (hypocalcemia, nausea, vomiting). For instance, the existence of an end-stage renal disease is a condition that can lead to the risk of long QT interval in patients with cinacalcet (36). Apart from this particular condition, previous reports suggested that cinacalcet could be an effective therapeutic option to normalize hypercalcemia either in FHH-2 (37,38) or in FHH-3 patients (8). We consider that, in certain circumstances, the use of cinacalcet can be indicated on an individual basis, but raised the following question: how could we be sure that cinacalcet treatment is effective in these patients? Based on *in vitro* studies, the majority of inactivating *CASR* mutations seems to be efficiently targetable by cal cimimetic, however there is no data on the I555T variant (10). Clinically, we observed that the I555T *CASR* variant strongly affects the sensor activity of the CaSR with a significant shift of the set-point for blood calcium concentration as highlighted by high serum level of calcium in all patients. Moreover, I555T pathogenic variants have been previously reported in two clinical cases of severe hypercalcemia, one in a neonate (18), and the other one, in a 32 yo patient who had serum calcium of 3.39 mmol/l and at least, 3 out of his 6 first degree relatives with serum calcium ≥ 2.75 mmol/l (21). While there was no information regarding ECG data in both reports, together with our family, these observations suggest that the residue 555 may be crucial for the CaSR transduction. Another variant involving the amino acid 555 with valine instead of isoleucine (c.1663AG p.Ile555Val) was identified in a patient with a possible FHH phenotype, however no accurate clinical or biochemical data were available (22). Human CaSR is expressed on the cell surface as a constitutive homodimer(14) and the structural modelling of mutant CaSR we designed suggests that I555T variant could directly or indirectly disrupt Ca^2+^ binding to this site, influence receptor dynamics and communication between LB1/LB2 and transmembrane domains (via the CRD). *In vitro* characterization of Ile555Thr-CaSR demonstrates that expression of this CaSR variant at the plasma is unaltered, but its intracellular calcium responses to alterations in extracellular calcium concentrations are markedly reduced, and consistent with it being a loss-of-function mutation, as reported for other FHH-1 associated CaSR mutations. Furthermore, treatment of Ile555Thr CaSR-expressing cells with cinacalcet rectifies these signaling defects. These findings provide evidence that the Ile555Thr variant results in impaired CaSR function. While positive, the acute test using cinacalcet in our study could reflect the action of the cinacalcet upon the normal wild-type CaSR rather than a real sensitivity of the mutant CaSR.

The acute cinacalcet test is a simple approach that can be used in clinical practice to ensure that the patient is likely to benefit to the treatment and can be eventually complemented by *in silico* prediction models. This test is based on the oral intake of 30 mg cinacalcet on the morning, and the assessment of serum calcium, phosphate and PTH levels every 2 hours for 8 hours. To consolidate its validity, conducting this functional test in healthy volunteers would be important for future investigations. We show that cinacalcet therapy allows normalization of the plasma calcium and the electrocardiogram, ultimately reducing the risk of sudden death in young patients; no adverse effects occurred during cinacalcet treatment in both children, but the mother had to discontinue the therapy because of nausea.

In conclusion, we show that screening for a short QT interval should systematically be part of the evaluation of FHH-1 patients. This study gives a new impetus towards the need to update the management recommendations for FHH patients, as suggested by others(9,10), taking into account more severe biochemical phenotypes with the determination of an individual cardiac-risk assessment and benefit-risk ratio of initiating cinacalcet therapy. A cinacalcet test could select patients who are likely to benefit from the treatment.

## Supplementary Material

Supplementary material

## Figures and Tables

**Table 1:** Clinical, Biological, and Imaging features at baseline and during Treatment BMD: Bone Mineral Density

Variable	Mother	Daughter					Son			Reference range
Sex	Female	Female					Male			
Age at diagnosis - yr	39	21					19			
Weight - kg	57	42					70			
Size - cm	167	153					168			
Body surface - m^2^	1.63	1.341					1.828			
Associated clinical features	None	Heart palpitations while resting					None			
**Serum measurements and therapeutic intervention**		**Cinacalcet Therapy**					**Cinacalcet Therapy**			
	**Prestudy**	**Prestudy**	**M1**	**M2**	**M3**	**Prestudy**	**M1**	**M2**	**M3**	
Albumin-adjusted Calcium - mmol/liter	2.8	2.95	2.25	2.4	2.47	2.88	2.65	2.60	2.55	2.15 - 2.50
Phosphate - mmol/liter	0.77	0.65	0.97	1.05	1.09	0.85	0.94	1.20	1.26	0.81 - 1.45
Creatininemia (μmol/l)	58	48.7	52	54	56	67.4	79	81	76	45 - 84
Creatinine clearance (ml/min/1.73m^2^)	96	135	131	130	128	136	125	121	121	> 90
Vitamine D (nmol/l)	42.7	62	115	85	52.5	65	155	90	63	75 - 250
Magnesium - mmol/liter	0.82	0.93	0.97	0.86	0.99	0.76	0.87	0.90	0.84	0.66 - 1.07
Parathyroid hormone - ng/liter	98	73.6	32	57	43	58.5	13	13	27	15.1 - 65.1
24h-calciuria (mmol/24h/1.73m^2^)	4.8	4.6	6.2	3.7	6.9	4.1	4.3	4.4	4	2.5 - 7.5
Urinary calcium-to-creatinine clearance ratio	0.008	0.009	0.028	0.012	0.006	0.006	0.012	0.008	0.008	> 0.02
**Imaging parameters**										
Neck US	Negative	Negative					Negative			
Sestamibi scanning	Left P3	Negative					Negative			
Kidney US	Negative	Negative					Negative			
BMD	Z-score (L1-L4): -0.9SD Z-score (Hip): 0.4SD	Z-score (L1-L4) : -0.4SD Z-score (Hip): 0.4SD					Z-score (L1-L4): 0.1SD Z-score (Hip): 2.1SD			
**ECG records**										
QT interval - milliseconds	349	340	-	-	420	340	-	-	400	> 340
**Transthoracic Echocardiography**	Normal	Normal			Normal					

## Data Availability

Original data generated and analyzed during this study are included in this published article or in the data repositories listed in References
