# Supplementary material for: Cinacalcet reverses short QT interval in Familial Hypocalciuric Hypercalcemia type 1
Source: J Clin Endocrinol Metab. Author manuscript; Available in PMC 2024 Jan 21. (PMC7615553; doi:10.1210/clinem/dgad494)

Supplementary Figure 1. Probands of family. Family tree and DNA sanger sequencing results showing the c.1664T>C *CASR* variant and, as a comparison the wild type sequence (CTRL).

Supplementary Figure 2. Schematic cartoon representation of human CaSR mutant I555T. Models for the I555T mutant were generated using the recent cryo-EM structure of wild-type human CaSR in the active form as a template (pdb code 7DTT). Human CaSR is a homodimer composed of an extracellular domain and a transmembrane domain. The extracellular domain consists of two lobe-shape domains (LB1 and LB2) and a cysteine-rich domain (CRD). The two protomers of human CaSR are shown as blue and green cartoon representations, respectively. The I555T mutation occurs at the dimerization interface, at the junction between CRD and LB2. Residue T555 is shown as coloured spheres. The flexible loop containing residue 555 is highlighted in orange. Calcium (Ca^{2+}) ions are represented as green spheres. The binding site of phenylalkylamine cinacalcet (mimpara) in the transmembrane domain is shown as a red surface based on previously reported docking models. The right panel correspond to the same representation after a 90° rotation along y axis.

Supplementary Figure 3. Schematic cartoon representation of WT human CaSR. 3D structure of active wild-type human CaSR was recently solved by cryo-EM structure (pdb code 7DTT). Residue I555 is shown as coloured spheres. See supplementary Figure 2 for a detailed description of the representation.

Supplementary Figure 4. Superposition of WT and I555T mutant human CaSR. Superposition of the 3D structure of active wild-type human CaSR (red) and T555 mutant. See Supplementary Figure 2 for a detailed description of the representation.

Supplementary Figure 5. Superposition of WT and I555T mutant human CaSR. Same as Supplementary Figure 4 with a focus on residue 555.

Supplementary Motion. Animation highlighting the location of residue 555 during human CaSR activation. Homology models of Human CaSR I555T mutant were built with MODELLER v9.24 for both inactive and active forms using known 3D structures as templates (pdb codes 7DTW and 7DTT, for the inactive and active forms, respectively). An *in house* script was used to generate a morphing animation between the two models. Residue 555 is located at the interface between the two chains of CaSR, therefore the change of hydrophobic isoleucine to polar threonine in position 555, could have an impact in the activation of human CaSR.

Supplementary Figure 6. Comparison of the CaSR Isoleucine (I) residue 555 between the species. Ensembl Data Sources (www.Ensembl.org) shows a highly conserved amino acid between species

Field Code Changed

Supplementary Figure 7. In silico analysis using the Polyphen-2 tool (http://genetics.bwh.harvard.edu/pph2/) of the I555T mutant human CaSR.

Supplementary Figure 8. A) Sanger sequencing of mutagenized pcDNA5/FRT-*CASR* expression construct confirming successful mutagenesis of the thymine (T) residue at position c.1664 (highlighted) within exon 6 of the *CASR* to a cytosine (C) B) Predicted outcome of mutagenesis causing an alteration in amino acid 555 from isoleucine (Ile) to threonine (Thr).

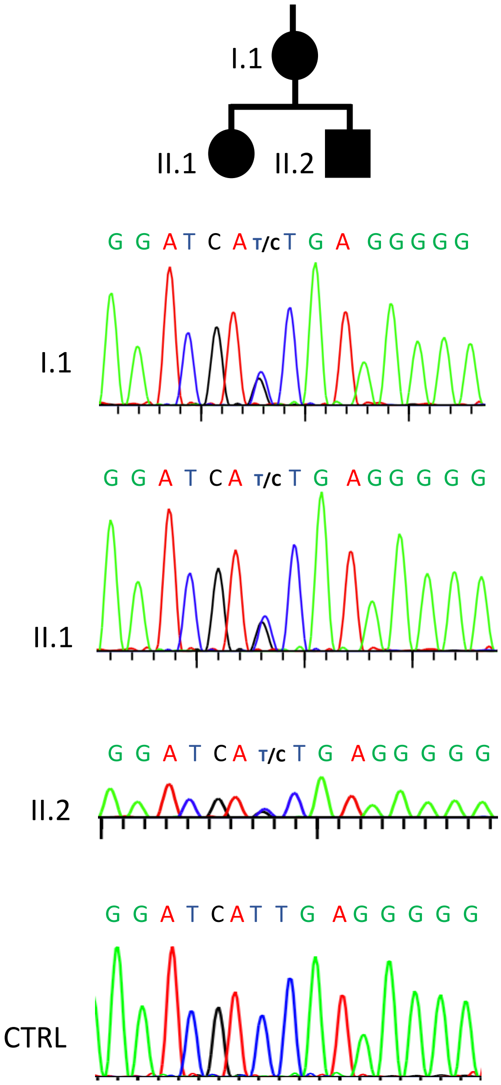

Supplementary Figure 1

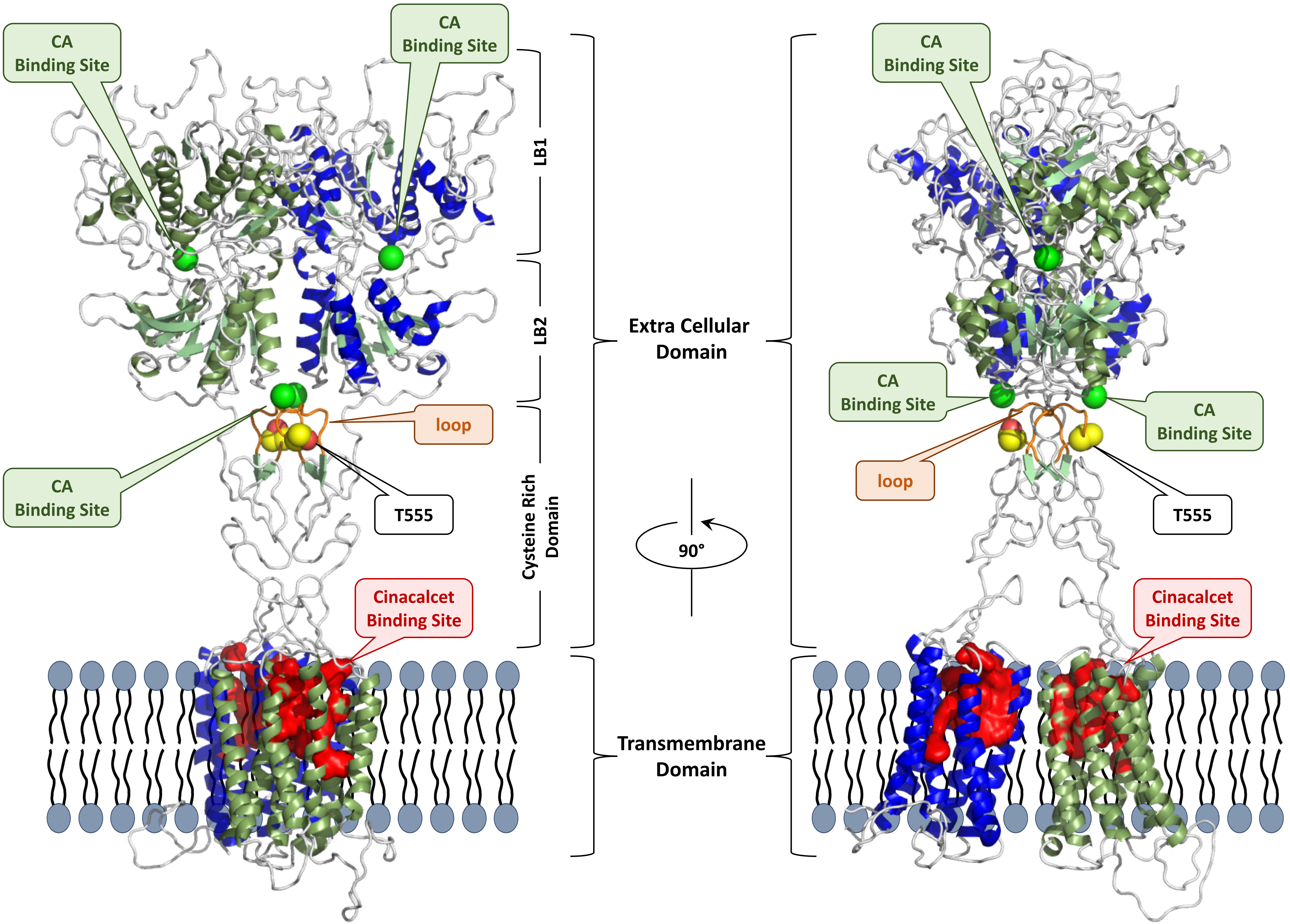

Supplementary Figure 2

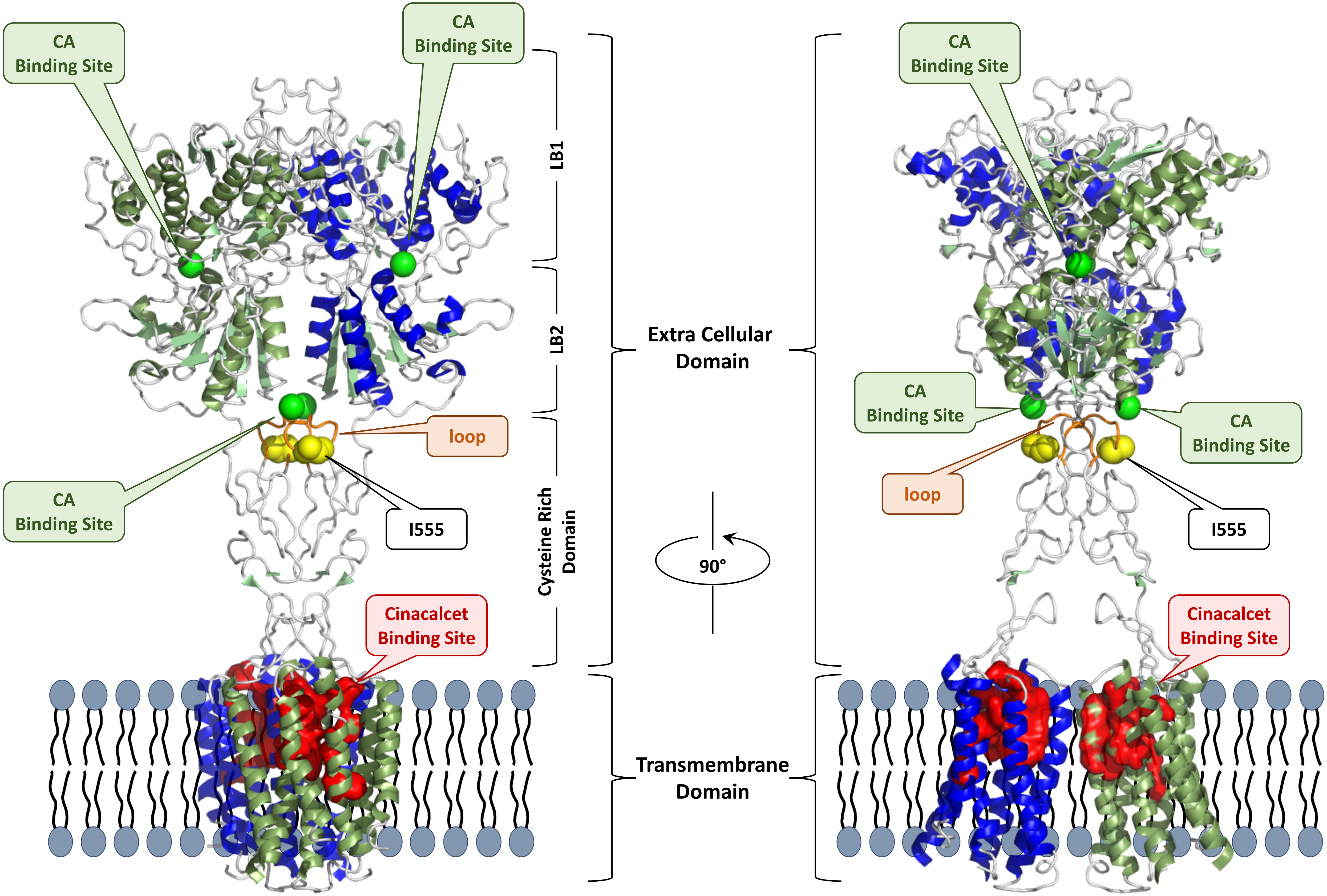

Supplementary Figure 3

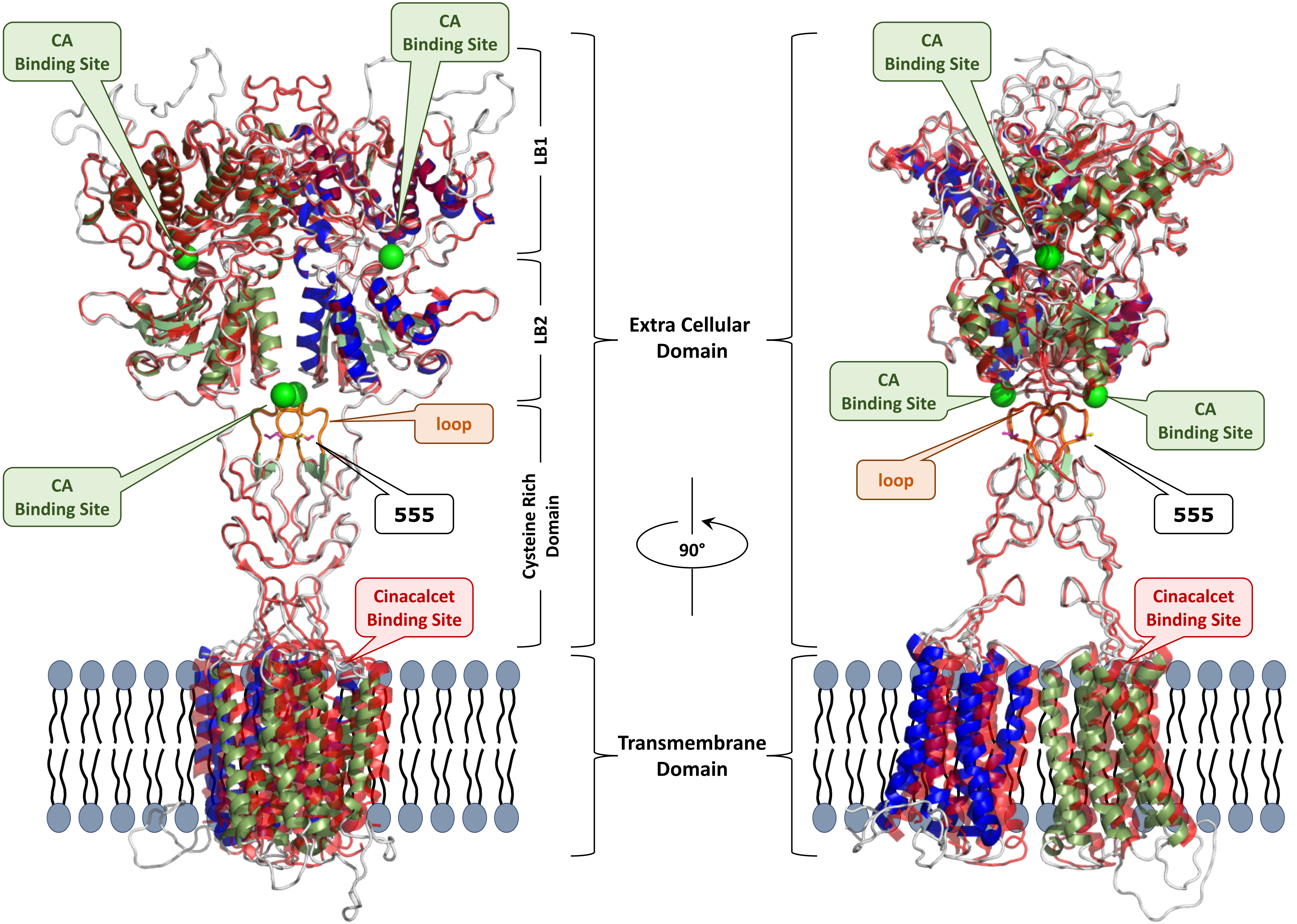

Supplementary Figure 4

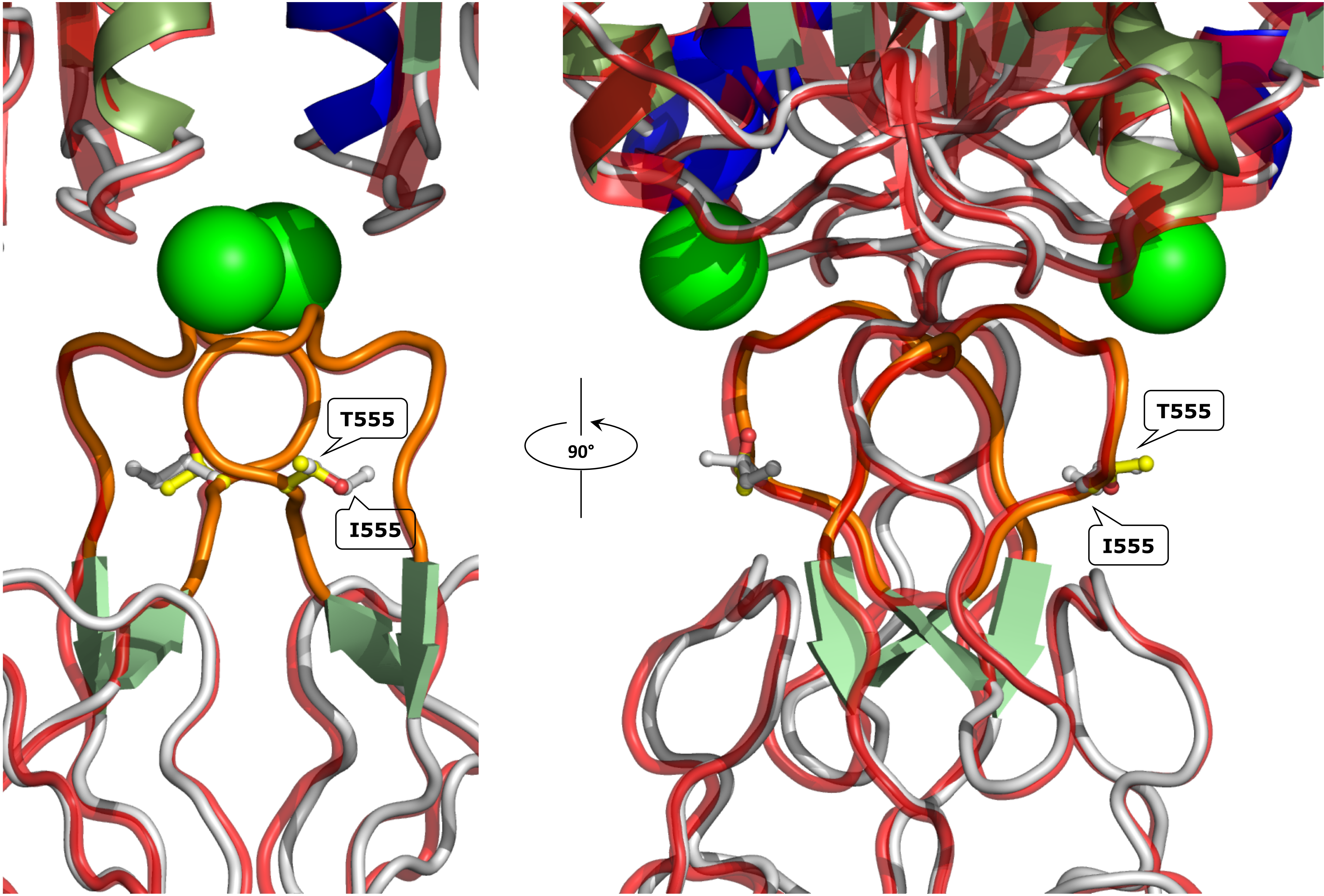

Supplementary Figure 5

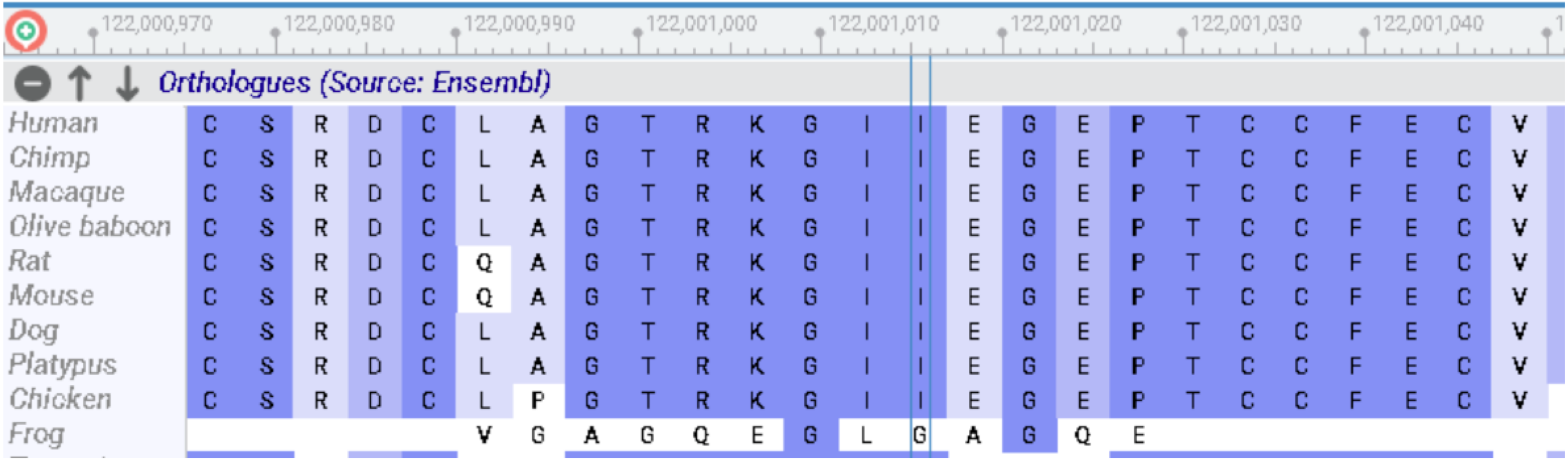

Supplemental Figure 6

PolyPhen-2 report for Human I555T

Query

Protein Acc	Position	AA_1	AA_2	Description
Human	555	I	T	CASR

Results

Prediction/Confidence *PolyPhen-2 v2.2.3r406*

HumDiv

This mutation is predicted to be **PROBABLY DAMAGING** with a score of **0.998** (sensitivity: **0.27**; specificity: **0.99**)

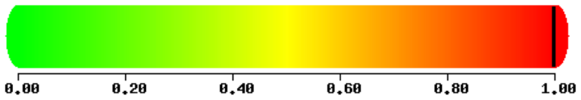

HumVar

This mutation is predicted to be **PROBABLY DAMAGING** with a score of **0.966** (sensitivity: **0.61**; specificity: **0.93**)

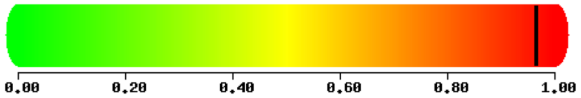


```
QUERY               ILWSGFSRE----------VPFSNCSRDCLAGTRKGIIEGEPTCCFECVECPDGEYSDET--DASACNKCPDDFWS
sp|G3UX06#1         ILWSGFSRE----------VPFSNCSRDCQAGTRKGIIEGEPTCCFECVECPDGEYSGET--DASACDKCPDDFWS
sp|F6SCD2#1         ILWSGFSRE----------VPFSNCSRDCLAGTRKGIIEGEPTCCFECVECPDGEYSDET--DASACDKCPDDFWS
sp|Q9QY96#1         ILWSGFSRE----------VPFSNCSRDCQAGTRKGIIEGEPTCCFECVECPDGEYSGET--DASACDKCPDDFWS
sp|P48442#1         ILWSGFSRE----------VPFSNCSRDCQAGTRKGIIEGEPTCCFECVECPDGEYSGET--DASACDKCPDDFWS
sp|Q80ZA8#1         ILWSGFSRE----------VPFSNCSRDCQAGTRKGIIEGEPTCCFECVECPDGEYSGET--DASACDKCPDDFWS
sp|G1SN65#1         ILWSGFSREPLTSVHSVLQVPFSNCSRDCLAGTRKGIIEGEPTCCFECVECPDGEYSDET--DASACDKCPDDFWS
sp|G3WHI5#1         ILWSGFSRE----------VPFSNCSRDCLAGTRKGIIEGEPTCCFECVECPDGEYSDET--DASACDKCPDDSWS
sp|F6PW91#1         ILWSGFSRE----------VPFSNCSRDCLAGTRKGIIEGEPTCCFECVECPDGEYSDET--DASACDKCPDDSWS
sp|UPI000194B9FA#1  ILWGGFSKE----------VPFSNCSRDCLPGTRKGIIEGEPTCCFECVDCPDGEYSDET--DASACDKCPEDYWS
sp|G1NMI7#1         ILWSGFSKE----------VPFSNCSMDCLPGTRKGIIEGEPTCCFECVDCPDGEYSDET--DASACDKCPEDYWS
sp|F6UGD8#1         ILWSGFSRE----------VPFSNCSRDCLAGTRKGIIEGEPTCCFECVECPDGEYSGET--DASACDKCPDDSWS
sp|UPI000223F6EF#1  ILWSGFSRE----------VPFSNCSRDCLAGTRKGIIEGEPTCCFECVECPDGEYSGET--DASACDKCPDDSWS
sp|UPI0001F9B995#1  ILWSGFSKE----------VPFSNCSRDCQPGTRKGIIEGEPTCCFECVECTDGEISDET--DASACEKCAENFWS
sp|F1NHH3#1         ILWSGFSKE----------VPFSNCSRDCLPGTRKGIIEGEPTCCFECVDCPDGEYSDET--DASACDKCPEDYWS
```

Shown are 75 amino acids surrounding the mutation position (marked with a black box)

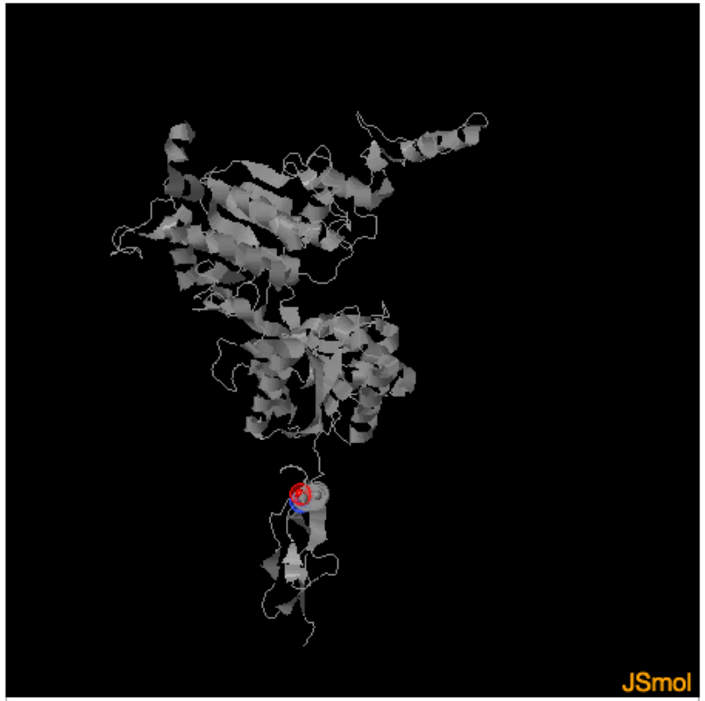

Zoom into mutation | Reset view | View size: + −

EntryID: 5K5T
ChainID: A
Residue: Ile555
Identity: 98.8%
Overlap: 53.6% (578 aa)

Supplementary Figure 7

A

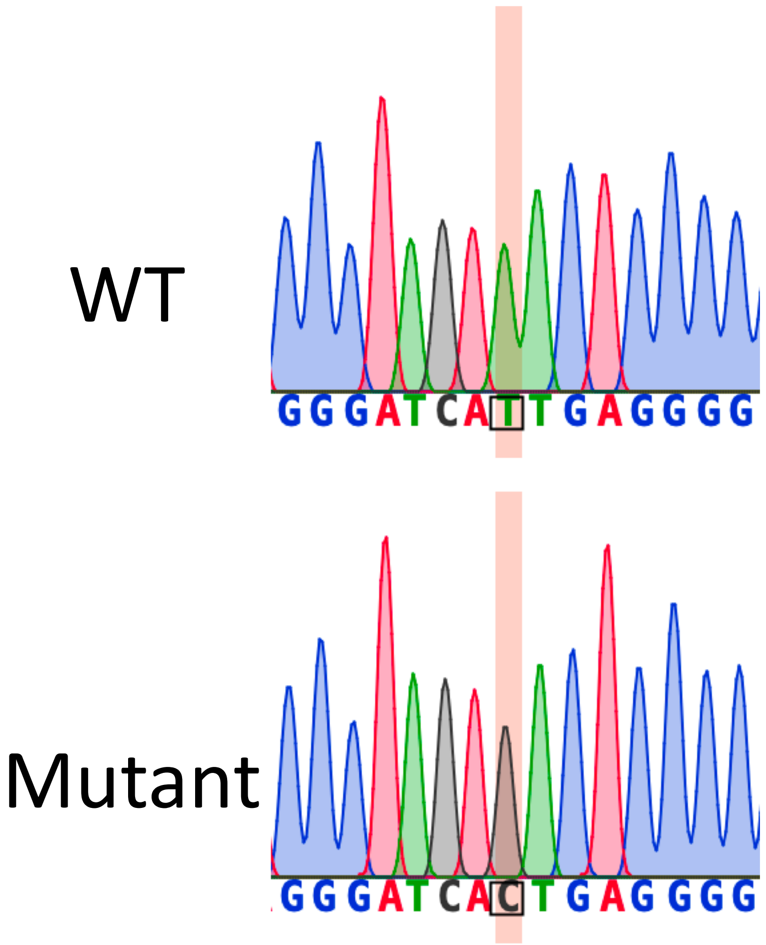

B

**Codon**	553	554	555	556	557
**WT**			Ile		
**Amino acid**	Gly	Ile		Glu	Gly
**Mutant**			Thr		
**WT**			T		
**Nucleotide**	GGG	ATC	A T	GAG	GGG
**Mutant**			C		

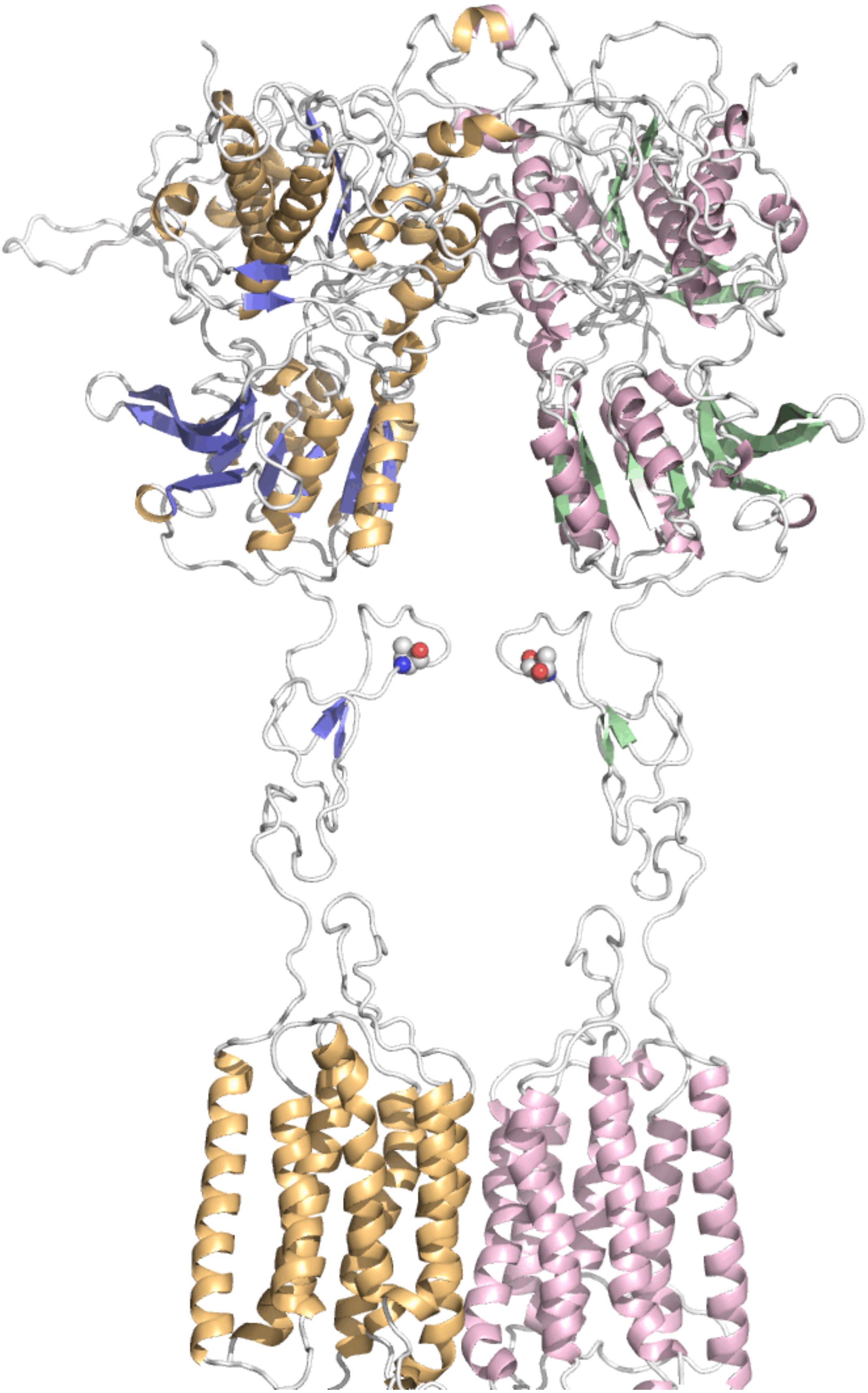

Supplement: Supplementary material [file EMS185262-supplement-Supplementary_material.pdf]
